# Psoralidin inhibits osteosarcoma growth and metastasis by downregulating ITGB1 expression via the FAK and PI3K/Akt signaling pathways

**DOI:** 10.1186/s13020-023-00740-w

**Published:** 2023-03-31

**Authors:** Shengwen Cheng, Senrui Liu, Bowen Chen, Chengcheng Du, Pengcheng Xiao, Xuefeng Luo, Li Wei, Yiting Lei, Chen Zhao, Wei Huang

**Affiliations:** The First Affiliated Hospital of Chongqing Medical University, Chongqing, 400016 China

**Keywords:** Osteosarcoma, Psoralidin, ITGB1, FAK, PI3K/Akt

## Abstract

**Background:**

*Psoralea corylifolia* is a medicinal leguminous plant that has long been used to treat various diseases. Psoralidin (PSO) is the main extract compound of *P. corylifolia* and exhibits antibacterial, antitumor, anti-inflammatory, antioxidant, and other pharmacological activities. PSO has demonstrated inhibitory effects in several cancers; however, its inhibitory effect on osteosarcoma has not been reported. This study aimed to evaluate the inhibitory effect of PSO on osteosarcoma and elucidate the underlying molecular mechanisms.

**Methods:**

Crystal violet, cell counting kit-8 (CCK8), and 5-Ethynyl-2′-deoxyuridine (EdU) staining assays were used to assess the inhibitory effect of PSO on the proliferation of 143B and MG63 osteosarcoma cells. Wound healing and Transwell assays were conducted to evaluate the effects of PSO on osteosarcoma cell migration and invasion. The cell cycle and apoptosis were analyzed using flow cytometry. To determine the possible molecular mechanisms, RNA-sequencing was performed and protein expression was analyzed by western blotting. The inhibitory effect of PSO on osteosarcoma in vivo was analyzed using a mouse model of orthotopic osteosarcoma and immunohistochemistry.

**Results:**

PSO inhibited osteosarcoma cell proliferation in a concentration-dependent manner, inhibited cell migration and invasion, and induced cell-cycle arrest and apoptosis. Mechanistically, PSO treatment significantly inhibited the focal adhesion kinase (FAK) and phosphatidylinositol 3-kinase (PI3K)/Akt signaling pathways by downregulating ITGB1 expression in both MG63 and 143B cells. Furthermore, we demonstrated that PSO restrained osteosarcoma growth in vivo.

**Conclusion:**

PSO may suppress osteosarcoma via the FAK and PI3K/Akt signaling pathways by downregulating ITGB1 expression.

**Supplementary Information:**

The online version contains supplementary material available at 10.1186/s13020-023-00740-w.

## Background

**Fig. 1:**
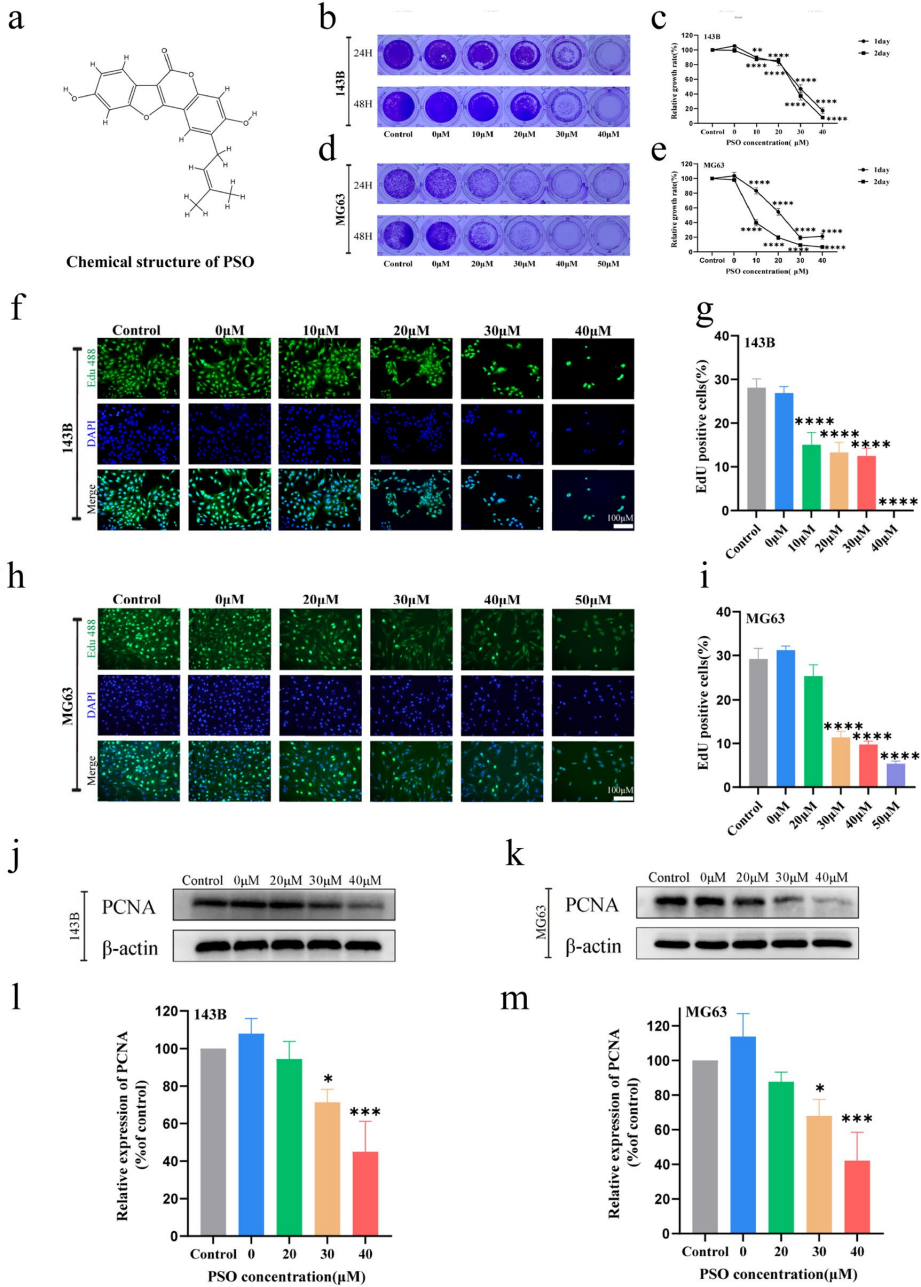
PSO inhibits OS cell proliferation in vitro. **a** Chemical structures of PSO. The effect of PSO on the proliferation of human OS cells was detected by crystal violet staining **b**–**e** and EdU staining **f**–**i**. Western blot analysis showed that PSO downregulated PCNA. PSO significantly inhibited the proliferation of OS cells **j**–**m** (*P < 0.05, ***P < 0.001, ****P < 0.0001 vs. the control group, n = 3)

*Psoralea corylifolia* is a traditional Chinese medicinal plant of the Leguminosae family [1]. Psoralidin (PSO), a coumestan derivative, is a compound extracted from *P. corylifolia* of the same class as psoralen and has various pharmacological activities, including anti-inflammatory, antioxidant, and antibacterial properties (Fig. 1a) [2–4]. For example, in prostate cancer cells, PSO effectively inhibited cell proliferation and promoted apoptosis through autophagy [5]. In breast cancer cells, PSO significantly downregulated Notch1 signaling and inhibited growth [6]. In colon cancer cells, it induced cell-cycle arrest and apoptosis [7]. Further, PSO is known to have a protective effect on normal tissues. At present, most tumors are treated using radiotherapy and chemotherapy. However, these therapies often have negative side effects, causing damage to normal organ tissues. PSO reportedly has a protective effect against doxorubicin-induced cardiotoxicity [8] and may protect against lung tissue injury after radiotherapy [9]. Its multi-target anticancer activity and potential organ-protective function make it a promising antitumor drug candidate.

Osteosarcoma (OS) is the most common primary bone malignancy in children, adolescents, and young adults, with a high tendency for local invasion and distant metastasis [10]. Current therapies for OS include neoadjuvant chemotherapy, surgery, and adjuvant (post‐surgery) chemotherapy [11, 12]. Substantial progress has been made in OS treatment in recent years, and the five-year survival rate for patients with the local disease has increased to approximately 65% [13, 14]. However, in cases of metastatic lesions, the five-year survival rate remains dismal, at less than 20% [15]. Tumor migration and invasion are key factors involved in tumor metastasis and are closely related to poor patient prognosis [16]. Therefore, it is of great significance to develop adjuvant drugs that can effectively inhibit OS.

In this study, we investigated the potential antitumor effects and molecular mechanisms of PSO in OS cell lines. Our results suggest that PSO may inhibit the initiation and progression of OS via the focal adhesion kinase (FAK) and phosphatidylinositol 3-kinase (PI3K)/Akt signaling pathways by downregulating ITGB1 expression.

## Methods

### Cell culture and drug preparations

Human OS cell lines 143B and MG63 were purchased from the American Type Culture Center (Manassas, VA, USA). Dulbecco’s modified Eagle’s medium (DMEM), fetal bovine serum (FBS), and penicillin–streptomycin were purchased from Gibco (Carlsbad, CA, USA). Psoralidin (20 mg, 99% purity), Cell Counting Kit-8 (CCK8), and dimethyl sulfoxide (DMSO) were purchased from China MCE. Crystal violet staining solution was purchased from Beijing Solarbio Science & Technology, and 5-Ethynyl-2′-deoxyuridine (EdU) 488 and BCA protein concentration kits from Beyotime Biotechnology. Transwell assay kits were purchased from Guangzhou Jet Bio-Filtration, and Matrigel from BD Biosciences, USA. To create a PSO stock solution, 20 mg of PSO was dissolved in 595 µL of DMSO to make a final concentration of 100 mM. The stock solution was stored at –20 °C and diluted in fresh medium before use.

### CCK8 assay

MG63 and 143B cells were collected at the logarithmic growth phase, digested, and counted. Next, they were mixed with 10% FBS, seeded in 96-well culture plates at a density of 5 × 10^3^ cells/well, and incubated for 24 h. After cell adhesion, different concentrations of PSO were added, and the plates were incubated for 24 h or 48 h. The cells were then incubated in 10% CCK8 liquid diluted in normal medium at 37 °C for 2 h. The spectroscopic absorbance at 450 nm in each well was measured using an automated plate reader. To investigate the anti-proliferative effect of PSO on OS cells, 143B, and MG63 cells were treated with 0–50 µM PSO, and cell viability was measured at 24 h and 48 h. Control cells were treated with DMSO. After 24 h of treatment, the half-maximal inhibitory concentration (IC50) of PSO in the OS cells was calculated (IC50, MG63 = 31.44 µM, 143B = 29.52 µM). Based on these data, we selected 10, 20, 30, and 40 µM for 143B cells and 20, 30, 40, and 50 µM for MG63 cells as working PSO concentrations for subsequent experiments.

### Crystal violet assay

MG63 and 143B cells were seeded in 96-well plates at a density of 5 × 10^3^ cells/well, incubated overnight, and treated with different concentrations of PSO for 24 h or 48 h. Subsequently, the cells were stained with crystal violet. The crystal violet in the plates was completely dissolved with a 20% acetic acid solution. The OD at 590 nm, which approximates the wavelength of maximum absorbance for the enzyme marker, was measured in each well to quantify cell proliferation activity.

### EdU cell staining assay

MG63 and 143B cells were cultured in 6-well plates (2 × 10^5^ cells/well) at 37 °C in the presence of 5% CO_2_ for 24 h. Then, the EdU solution was added to the wells and the plates were incubated at 37 °C for another 4 h. The cells were fixed with 4% paraformaldehyde for 20 min and then permeated with 0.3% Triton-100 for 15 min. They were then incubated with 500 µL of click reaction solution for 30 min. Nuclei were counterstained with 4′,6-diamidino-2-phenylindole (DAPI), and the EdU/DAPI-positive rate was calculated. Images were acquired and observed using a fluorescence microscope (Nikon Clipstie, Japan).

### Wound healing assay

MG63 and 143B cells were seeded into 6-well plates and grown to confluence. The monolayer in each well was scratched with a 200 μL pipette tip and then washed three times with PBS to remove cell debris and suspended cells. Next, fresh medium with different concentrations of PSO was added to each well. The scratched area was photographed at various time points (6, 12, and 24 h), and the recovery area was calculated using the ImageJ software.

### Cell migration and invasion assays

To measure cell invasion capacity, Matrigel was thawed at 4 °C and diluted in serum-free DMEM at a ratio of 1:8. The mixed solution was added to Transwell chambers containing 80 μL of medium/well and the plates were incubated at 37 °C in the presence of 5% CO_2_ for 4 h. Subsequently, 2.5 × 10^4^ cells of each OS cell line were respectively added to the upper Transwell chambers, and different concentrations of PSO were added to the lower chambers. After incubation for 24 h, the cells that had penetrated the Matrigel coating and migrated to the lower chamber were stained with crystal violet (0.1%) and photographed. To measure cell migration, the experiment was conducted under the same conditions, except without Matrigel in the upper chamber.

### Flow-cytometric analysis

Flow cytometry was used to determine the effects of PSO on OS apoptosis and the cell cycle. After treatment with PSO, cells were harvested and washed three times with PBS. To determine the apoptosis rate, cells were resuspended in 500 μL of PBS, stained using an Annexin V-FITC/PI double labeling staining kit according to the manufacturer’s instructions, and analyzed by flow cytometry. For cell-cycle assessment, cells were fixed with 70% ethanol at 4 °C overnight and analyzed using a flow cytometer (CytoFLEX, Beckman Coulter, Fullerton, CA, USA).

### Western blotting

OS cells were treated with different concentrations of PSO for 24 h and then lysed with RIPA buffer. The proteins were separated by sodium dodecyl sulfate–polyacrylamide gel electrophoresis and transferred to polyvinylidene difluoride membranes (0.45 μm). After blocking with 5% skim milk for 1 h and washing three times with Tris-buffered saline (TBS), the membranes were probed with primary antibodies at 4 °C overnight. After washing three times with TBS-Tween, the membranes were incubated with a secondary antibody (goat anti-rabbit/mouse IgG, 1:5000) at room temperature for 2 h. Protein bands were imaged and analyzed using a ChemiDoc MP Imaging System and Image Lab Software (Bio-Rad, Hercules, CA, USA). The following antibodies were used: anti-PCNA (ab92552, Abcam), anti-matrix metalloproteinase (MMP) 2 (ab92536, Abcam), anti-MMP9 (ab76003, Abcam), snail rabbit mAb (#3879, CST), bcl-2 rabbit mAb (#4223, CST), bax rabbit mAb (#41,162, CST), cleaved caspase-3 rabbit mAb (#9654, CST), β-actin rabbit mAb (#4970, CST), anti-FAK (ab40794, Abcam), anti-p-FAK (phospho Y397) (ab81298, Abcam), anti-PI3 kinase p85 alpha (ab191606, Abcam), anti-p-PI3 kinase p85 alpha (phospho Y607) (ab182651, Abcam), anti-Akt (ab179463, Abcam), and anti-p-Akt (phospho S472 + S473 + S474) (ab192623, Abcam).

### RNA-sequencing

143B and MG63 cells were treated with 30 μM PSO for 24 h. Total RNA was extracted using an RNA Extraction Kit (Thermo Fisher Scientific, Waltham, MA, USA). The RNA samples were sent to Beijing Qingke Biotechnology (Beijing, China) for RNA-sequencing, which was performed on a DNBSEQ-T7 sequencer to determine changes in mRNA expression.

### Kaplan–meier analysis

The GSE21257 OS microarray dataset was downloaded from the GEO database (https://www.ncbi.nlm.nih.gov/geo/). This dataset included 53 OS biopsy samples collected before chemotherapy, with metastatic status and time information (34 metastatic and 19 non-metastatic samples), and transition status and time information. Kaplan–Meier curves were constructed in the R package survminer (version 0.4.6) to analyze survival.

### Molecular docking

The core protein structures of the protein–protein interaction network were downloaded from the PDB database (http://www.rcsb.org/) and ligands and water molecules were removed with PyMOL. The core ingredients were downloaded as an SDF file from the PubChem database (https://pubchem.ncbi.nim.nih.gov) and converted into mol2 format using the OpenBabel software. Finally, the docking of the obtained protein structures (receptors) and active components (ligands) with PSO was predicted using AutoDock software, and the docking results were visualized using PyMOL software.

### Real-time PCR

Total RNA was isolated using TRIzol Reagent according to the manufacturer’s instructions, cDNA was synthesized, and qPCR was performed. The primer sequences are listed in Additional file 1: Table S1.

### Small interfering RNA transfection

Small interfering RNA (siRNA) transfection was performed according to the manufacturer's instructions described previously. The siRNA sequences used to knock down ITGB1 expression are mentioned in Additional file 1: Table S2.

### Orthotopic OS model establishment

BALB/c nude female mice (3–4 weeks old, 15–20 g) were purchased from Hunan SJA Laboratory Animal. After adaption for 1 week, 50 µL of 143B suspension (2 × 10^7^cells/mL) was injected into the proximal tibia of the mice. The animals were subsequently administered different doses of PSO (5, 10, or 20 mg/kg) or DMSO via oral gavage at two-day intervals. After three weeks of treatment, the animals were euthanized. Tumor volumes were calculated using the following formula: 0.5 × L × W^2^, where L and W represent the length and width of the tumor, respectively. For subcutaneous tumor measurement, the length (L), the longer dimension, and width (W), which is the shorter dimension perpendicular to the plane of the length and parallel to the plane of the animal's body, were measured using a caliper.All animal experiments were approved by the Animal Care and Use Organization Committee IACUC of Chongqing Medical University.

### Hematoxylin and eosin staining and immunohistochemistry

Tumor tissues were collected, fixed in 4% paraformaldehyde for 24 h, and washed with PBS. The fixed tissue samples were embedded in paraffin, dewaxed, and stained with hematoxylin and eosin (H&E). Immunohistochemical staining of tumor sections was performed using antibodies against proliferating cell nuclear antigen (PCNA; 1:100), bax (1:100), and MMP2 (1:100). All sections were imaged using a light microscope at 40 × and 100 × magnifications.

### Statistical analysis

The SPSS 22.0 software package (IBM Corp., Armonk, NY, USA) was used for statistical analysis. The in vitro experiments were repeated three times, and the in vivo experiment was repeated five times. To test for differences among multiple groups, one-way analyses of variance were performed, and Tukey’s test was used for between-group comparisons. The experimental data are expressed as mean ± SD, and *P* < 0.05 was considered statistically significant.

## Results

### PSO inhibits OS cell growth

**Fig. 2:**
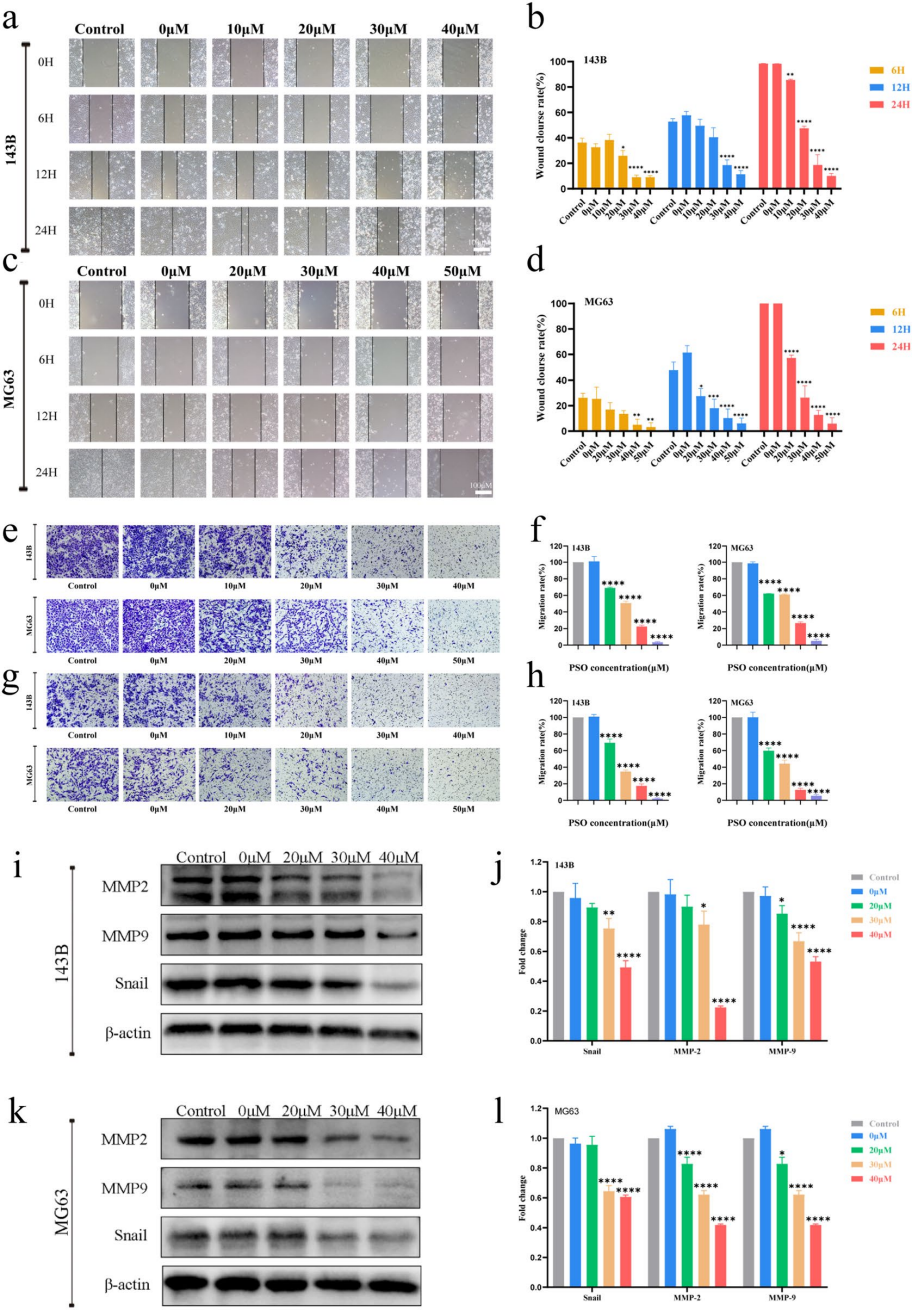
PSO inhibits the migration and invasion of OS cells. The effect of PSO on the migration ability of human OS cells was detected by wound healing (**a**–**d**, × 100) and Transwell (e–f, crystal violet staining, × 100) assays. The effect of PSO on the invasive ability of human OS cells was detected by Transwell assay (**g**–**h**, crystal violet staining, × 100). The effect of PSO on the protein expression of MMP-2, MMP-9, and snail was assessed by western blotting **i**–**l**. PSO significantly inhibited the migration and invasion of OS cells (*P < 0.05, **P < 0.01, ***P < 0.001, ****P < 0.0001, vs. the control group, n = 3)

The CCK-8 assay confirmed the inhibitory effect of PSO on the OS cell lines (Additional file 1: Fig. S1a, *P* < 0.05). Crystal violet staining results showed that PSO inhibited OS cell proliferation in a dose-dependent and time-dependent manner. In addition, EdU staining results indicated that with an increase in the PSO concentration, the proliferation rate of OS cells decreased significantly (Fig. 2b–i, P < 0.05). In addition, PSO reduced the protein expression of PCNA, a recognized marker used to evaluate OS growth (Fig. 2j–m, P < 0.05). Finally, we found that the range of safe concentration of PSO in normal cells (HK2 97.9 μM, LO2 221.6 μM) is higher than that in OS cells (Additional file 1: Fig. S1b, *P* < 0.05). Based on these results, we conclude that PSO effectively inhibits the proliferation of human OS cells.

### PSO inhibits OS cell migration and invasion

**Fig. 3:**
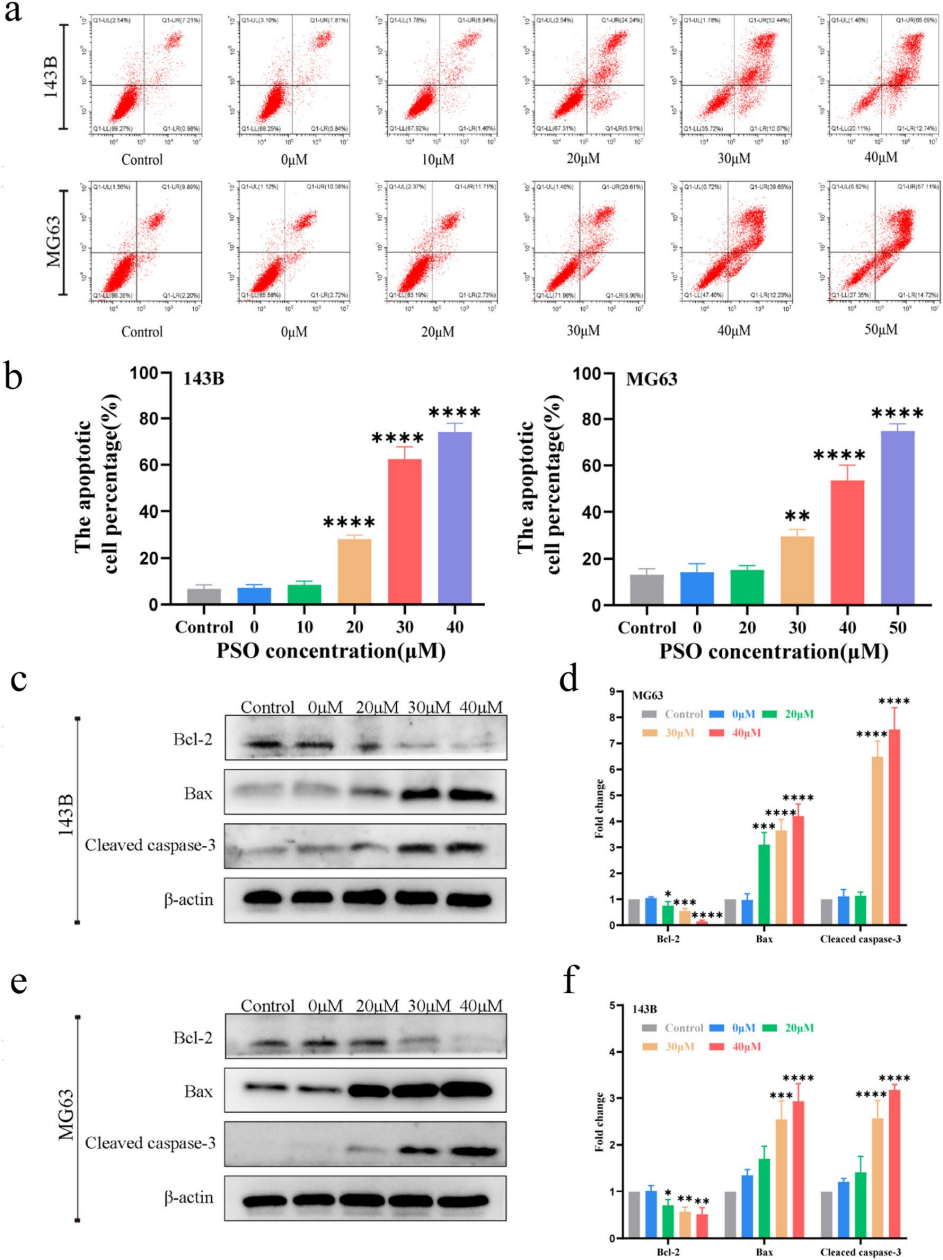
**a**–**b** The effect of PSO on the apoptosis of human OS cells was detected by flow cytometry. **c**–**f** The expression levels of the apoptosis-related proteins Bax, Bcl-2, and cleaved caspase 3 were detected by western blotting. PSO-induced OS cell-cycle arrest. PSO significantly promoted apoptosis in OS cells (*P < 0.05, **P < 0.01, ***P < 0.001, ****P < 0.0001, vs. the control group, n = 3)

The effect of PSO on the metastatic ability of OS cells was investigated by wound healing and Transwell assays. The wound healing assays showed that PSO inhibited the migration ability of OS cells, as indicated by a reduced wound healing rate (Fig. 2a–d, P < 0.05). The Transwell migration assays further demonstrated that PSO inhibited the migration of OS cells compared to the control treatment (Fig. 2e–f, P < 0.05). Further, PSO inhibited the invasive potential of OS cells compared to the control treatment as indicated by a Transwell invasion assay (Fig. 2g–h, P < 0.05). Epithelial-mesenchymal transition (EMT) and MMPs play key roles in tumor metastasis [17, 18]. To this end, western blotting results showed that PSO significantly reduced the expression of the EMT transcription factor, Snail, along with MMP2 and MMP9 (Fig. 3i–l, P < 0.05). Together, these in vitro experiments demonstrate that PSO inhibits OS cell migration and invasion.

### PSO induces cell-cycle arrest at the G0/G1 phase and promotes apoptosis in OS cells

Cell-cycle arrest and apoptosis induction are two major causes of cell proliferation inhibition. We used flow cytometry to investigate whether PSO had any effect on the cell cycle in OS cells. PSO treatment increased the number of OS cells in the G0/G1 phase (Additional file 1: Fig. S2a–d, *P* < 0.05). Quantification of the apoptotic rate of 143B and MG63 cells by flow cytometry revealed that PSO treatment significantly increased the apoptotic rate in both cell lines (Fig. 3a–b, P < 0.05). Western blot analysis results showed that the protein level of the anti-apoptotic factor Bcl-2 was decreased, whereas that of the pro-apoptotic factor Bax was increased after PSO treatment. In addition, the level of cleaved caspase-3, a classical marker of apoptotic activation, was increased by PSO treatment (Fig. 3c–f, P < 0.05). Taken together, these results indicate that PSO treatment induces cell-cycle arrest and apoptosis in OS cells.

### PSO downregulates ITGB1 expression and attenuates the FAK and PI3K/Akt signaling pathways

**Fig. 4:**
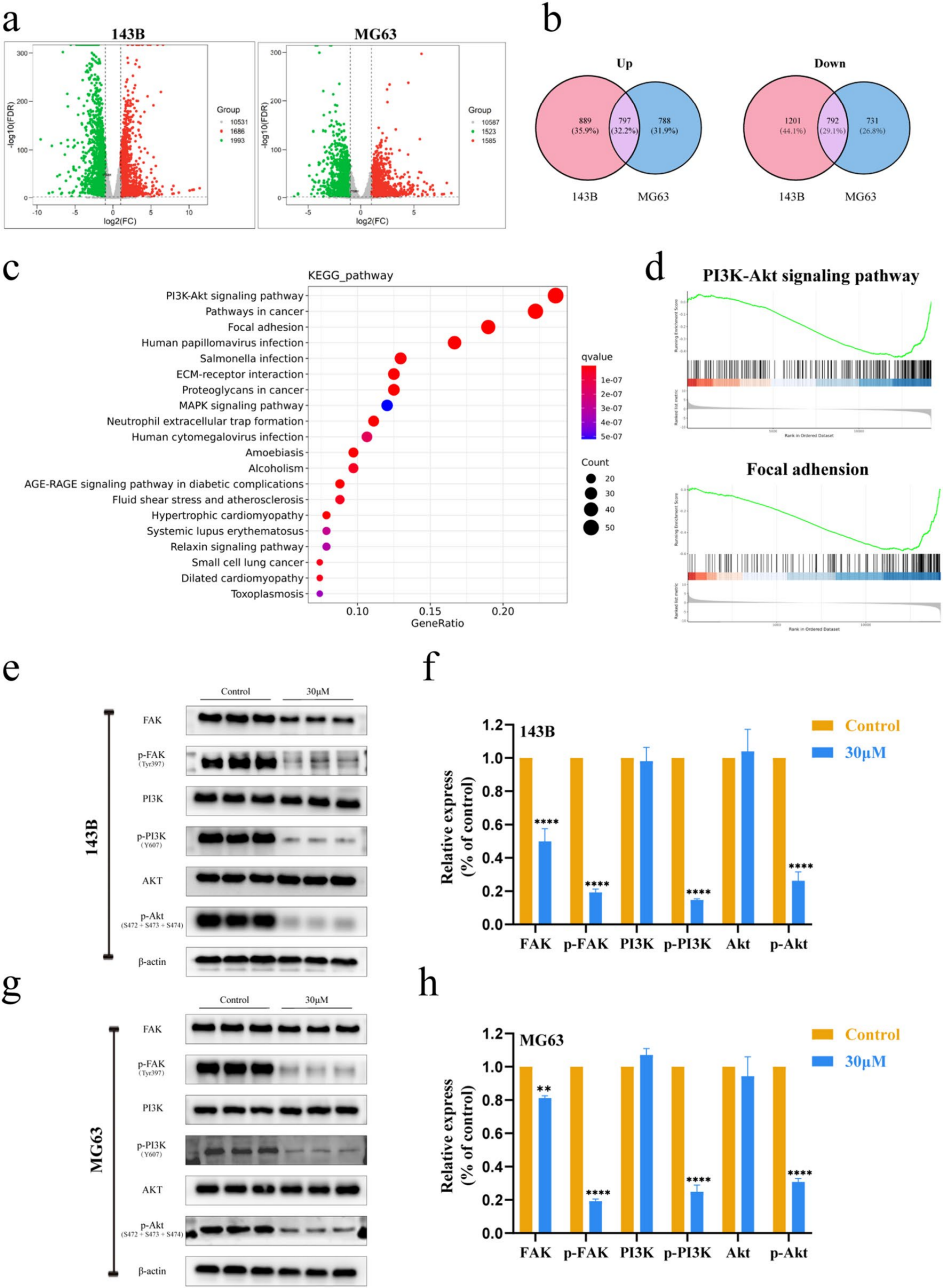
OS cells were treated with 30 μM PSO for 24 h, and the global changes in gene expression were analyzed by RNA-sequencing. **a**–**b** Volcano maps showing differentially expressed genes in OS cells treated with PSO compared to untreated control cells. **c** Altered signaling pathways in PSO-treated OS cells according to KEGG enrichment analysis. **d** GSEA enrichment plots for PSO-treated OS cells. **e**–**h** Western blotting analysis of FAK, p-FAK, PI3K, p-PI3K, AKT, and p-AKT levels in OS cells treated with PSO (*P < 0.05, **P < 0.01, ***P < 0.001, ****P < 0.0001, vs. the control group, n = 3)

To explore the possible molecular mechanisms underlying the antitumor effect of PSO, RNA was extracted from 143B and MG63 cells after 24 h of PSO treatment and subjected to RNA-sequencing analysis. Compared with control cells, 1686 upregulated and 1993 downregulated genes were found in 143B cells treated with PSO, and 1585 upregulated and 1523 downregulated genes in MG63 cells treated with PSO. Among them, 797 upregulated and 792 downregulated genes were common to both cell lines (Fig. 4a–b, Additional file 1: Fig. S3a). All genes identified were categorized into biological processes, cellular components, and molecular functions in GO analysis (Additional file 1: Fig. S3b).

**Fig. 5:**
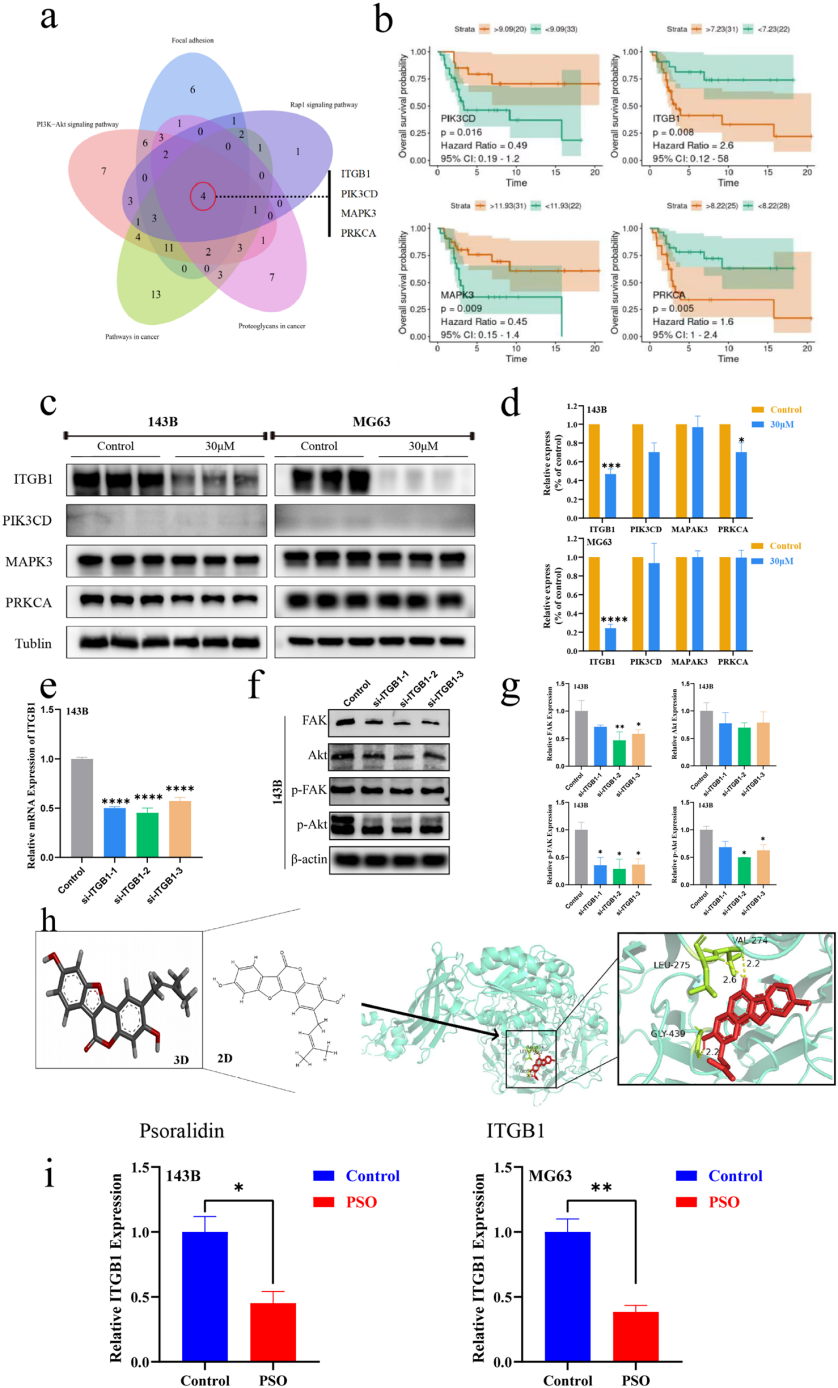
**a** Venn diagram of DEGs from selected top five pathways. **b** Kaplan–Meier curve representing the effects of different genes on the overall survival of OS patients. **c**–**d** Protein expression of ITGB1, PIK3CD, MAPK3, and PRKCA with or without PSO treatment. **e** After transfection with 100 nmol/L of the indicated siRNA or NC for 48 h, the expression of ITGB1 mRNA in 143B cells was analyzed by real-time PCR. **f**–**g** After transfection of siRNA into 143B cells, the expression of FAK, p-FAK, Akt and p-Akt pathway proteins was analyzed by western blot. **h** 2D and 3D molecular structures of PSO, stable complex, and docking pocket of PSO with ITGB1. (i) Relative expression of ITGB1 mRNA after PSO treatment (**P < 0.01, ****P < 0.0001, vs. the control group, n = 3)

Next, the differential expressed genes (DEGs) were subjected to KEGG pathway enrichment analysis (Fig. 4c). We took the intersection of genes showing the same gene expression change trends in both cell lines and screened only genes enriched with absolute normalized enrichment scores ≥ 1 and Q values ≤ 0.05 in gene set enrichment analysis (GSEA) (Fig. 4d). We then analyzed the total and phosphorylated levels of FAK and PI3K/Akt by western blotting. We found that the levels of FAK and the ratios of p-FAK: FAK, p-PI3K: PI3K, and p-Akt: Akt in 143B and MG63 cells were significantly decreased after 24 h of PSO treatment (Fig. 4e–h, Additional file 1: Fig. S4, *P* < 0.05), which was consistent with the RNA-sequencing results. After GSEA, Venn diagrams of the abovementioned top five pathways revealed four associated highly enriched DEGs: ITGB1, PIK3CD, MAPK3, and PRKCA. Among these, the expression of ITGB1, MAPK3, and PRKCA tended to decrease, whereas that of PIK3CD tended to increase after PSO treatment (Fig. 5a).

We downloaded the GSE21257 OS microarray dataset from the GEO database, including 53 OS biopsy samples. Using this dataset, Kaplan–Meier analysis was performed for the above four genes. The biopsy samples were divided into high-risk and low-risk groups based on patient outcomes, and all samples in the high-risk group from patients who developed OS metastasis within 5 years were compared with those in the low-risk group, in which patients did not develop OS metastasis. The Kaplan–Meier analysis showed that higher PIK3CD and MAPK3 expression was associated with better overall patient survival, whereas higher expression of ITGB1 and PRKCA was associated with lower overall survival. Overall, ITGB1 had the highest hazard ratio (Fig. 5b).

Then, the expression levels of the protein products of the four enriched DEGs were detected by western blotting. The results showed that 24 h after PSO treatment, ITGB1 levels were significantly decreased in both cell lines, the PRKCA levels were decreased only in 143B cells, and PIK3CD and MAPK3 levels were not significantly changed in both cell lines (Fig. 5c–d, P < 0.05). To further scrutinize whether ITGB1 can induce cellular proliferation and invasion through FAK and Akt signaling pathways, we analyzed the effects of obstructing FAK and Akt signal transduction by knocking down ITGB1 expression in 143B cells. Western blot analyses substantiated the efficacy of siRNA in inhibiting FAK, p-FAK, Akt, and p-Akt expression (Fig. 5e–g, P < 0.05). In addition, we found that Pyritegrin, an ITGB1 agonist, can partially restore the proliferation ability of OS cells treated with PSO (Additional file 1: Fig. S6, *P* < 0.05). A docking analysis of the above proteins was performed to elucidate their binding behavior with PSO. The molecular docking of ITGB1 with PSO showed that the protein formed a 2.6-Å hydrogen bond with amino acid residue LEU275, a 2.2-Å hydrogen bond with VAL274, and a 2.2-Å hydrogen bond with GLY439, forming a stable complex with PSO (Fig. 5h, Additional file 1: Fig. S5). RT-qPCR showed that the mRNA expression of ITGB1 was decreased after PSO treatment (Fig. 5i). Altogether, these results suggest that PSO may inhibit OS cell growth via the FAK and PI3K/Akt signaling pathways by downregulating ITGB1 expression.

### PSO inhibits tumor development in vivo

**Fig. 6:**
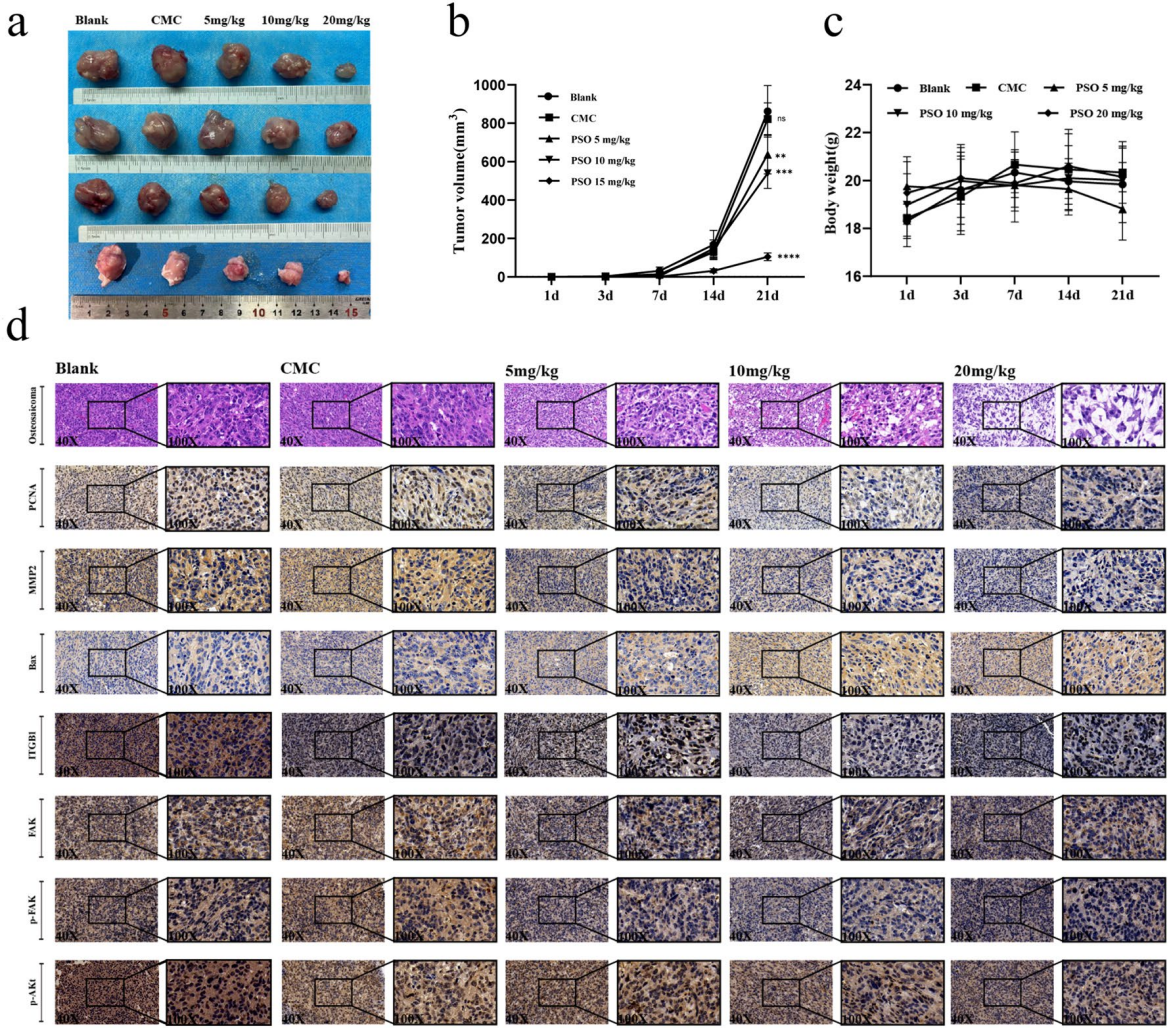
**a** PSO inhibits OS cell growth and metastasis in an OS mouse model. **b**–**c** The effects of PSO on tumor volume and mouse weight. **d** The effects of PSO on the tumor as detected by H&E staining and immunohistochemistry. PSO inhibited tumor development in vivo (vs. the CMC group, n = 4) (**P < 0.01, ****P < 0.0001, vs. the control group, n = 4)

We established an OS mouse model using 143B cells to assess the anti-OS therapeutic effect of PSO in vivo. The results indicated that PSO significantly inhibited tumor growth in a dosage-dependent manner (Fig. 6a, b). The body weight of the mice was not significantly reduced (Fig. 6c). H&E staining results revealed that while the tumor structure in the control group was clear and compact, in the PSO treatment group, we observed irregular morphology, elevated karyokinesis, and nuclear heterogeneity in the tumors. After administering PSO for a duration of three weeks, we conducted an in vivo safety evaluation of the drug by extracting normal tissue and blood from mice. H&E staining of the heart, liver, spleen, lung, and kidney tissues in each administration group showed no abnormal or pathological changes compared to the control group (Additional file 1: Fig. S7a). Moreover, we assessed the safety of the drug in vivo by analyzing liver and kidney function in tail orbital blood samples obtained from nude mice. (Additional file 1: Fig. S7b). In addition, we performed H&E staining of lungs after 3 weeks of treatment with PSO following injection of osteosarcoma cells through the tail vein. It was found that no significant lung texture disorder of the PSO-treated group compared to the control group, which suggested PSO effectively prevented the lung metastasis of osteosarcoma cells (Additional file 1: Fig. S8). Liver and kidney functions did not differ significantly in the PSO administration group compared to the control group. Hence, PSO did not cause obvious toxicity in vivo and showed good biological safety. Immunohistochemical staining showed that in the treatment group, the expression of PCNA, MMP2, ITGB1, FAK, p-FAK, and p-Akt was decreased, and that of Bax was increased (Fig. 6d, Additional file 1: Fig S9). These results confirm that PSO can inhibit tumor growth and metastasis in vivo by down-regulating ITGB1 expression via the FAK and PI3K/Akt signaling pathways.

## Discussion

OS is a highly malignant primary bone tumor. Despite the continuous improvement of clinical treatment, the survival rate of OS patients remains unsatisfactory, especially in patients with lung metastases [19, 20]. In addition, current anti-OS drugs such as ifosfamide, methotrexate, and cisplatin are prone to resistance. Moreover, although these drugs have anti-OS effects, most of them are highly cytotoxic to normal cells and can cause liver toxicity and kidney dysfunction [21–23]. Therefore, it is necessary to explore effective and safe drugs with relatively few side effects for the treatment of OS.

Psoralidin is the main extract compound of *P. corylifolia* [8] and has previously been shown to have significant inhibitory effects on various cancers. However, the exact mechanism of the anti-cancer effect of PSO remains unclear and may be multifactorial. Understanding the specific mechanism of action of PSO is essential for its use as a drug. It has been reported that PSO induces significant reactive oxygen species (ROS) generation, thus inhibiting cancer cell proliferation and inducing apoptosis through oxidative stress. Oxidative stress and ROS formation cause mitochondrial and lysosomal membrane damage and enhance membrane permeability. The mitochondrial membrane potential (ΔΨm) of cancer cells decreases after PSO treatment [24–26]. Other studies have revealed that suppression of the NF-κB and PI3K/Akt signaling pathways may be another mechanism of PSO. The PI3K-Akt pathway is a major signaling pathway involved in the oncogenesis of many cancer types [27]. In OS, the PI3K/Akt pathway is a common disorder-controlled carcinogenic signaling pathway [28]. PSO has been shown to inhibit the activity of Bcl-2 and induce the activities of Bax and caspase-3 via the PI3K/Akt pathway, thus reducing cell proliferation and promoting apoptosis [7, 29, 30]. In this study, we first established that PSO effectively inhibits OS cell proliferation, migration, and invasion in vitro and then demonstrated that it promotes OS cell apoptosis and inhibits OS tumor growth in vivo by downregulating ITGB1 expression via the FAK and PI3K/Akt signaling pathways.

Apoptosis is an important homeostatic mechanism involving the activation, expression, and regulation of a series of genes, and the regulation of tumor cell apoptosis has potential therapeutic significance [31]. Therefore, inducing tumor cell apoptosis is one strategy in the development of anticancer drugs. Using flow cytometry, we confirmed that PSO significantly increased the apoptosis rate of OS cells. Caspase-3 is the main executor of apoptosis and plays a role in the early stage [32]. Bcl-2 family proteins are key regulators of mitochondria-mediated apoptosis and include antiapoptotic members, such as Bcl-2, and proapoptotic members, such as Bax [33]. We found that PSO significantly increased the apoptosis rate of OS cells and promoted the protein levels of Bax and cleaved caspase-3, while downregulating Bcl-2 levels. The pathways via which PSO was found to promote OS cell apoptosis are consistent with those in previous studies.

**Fig. 7:**
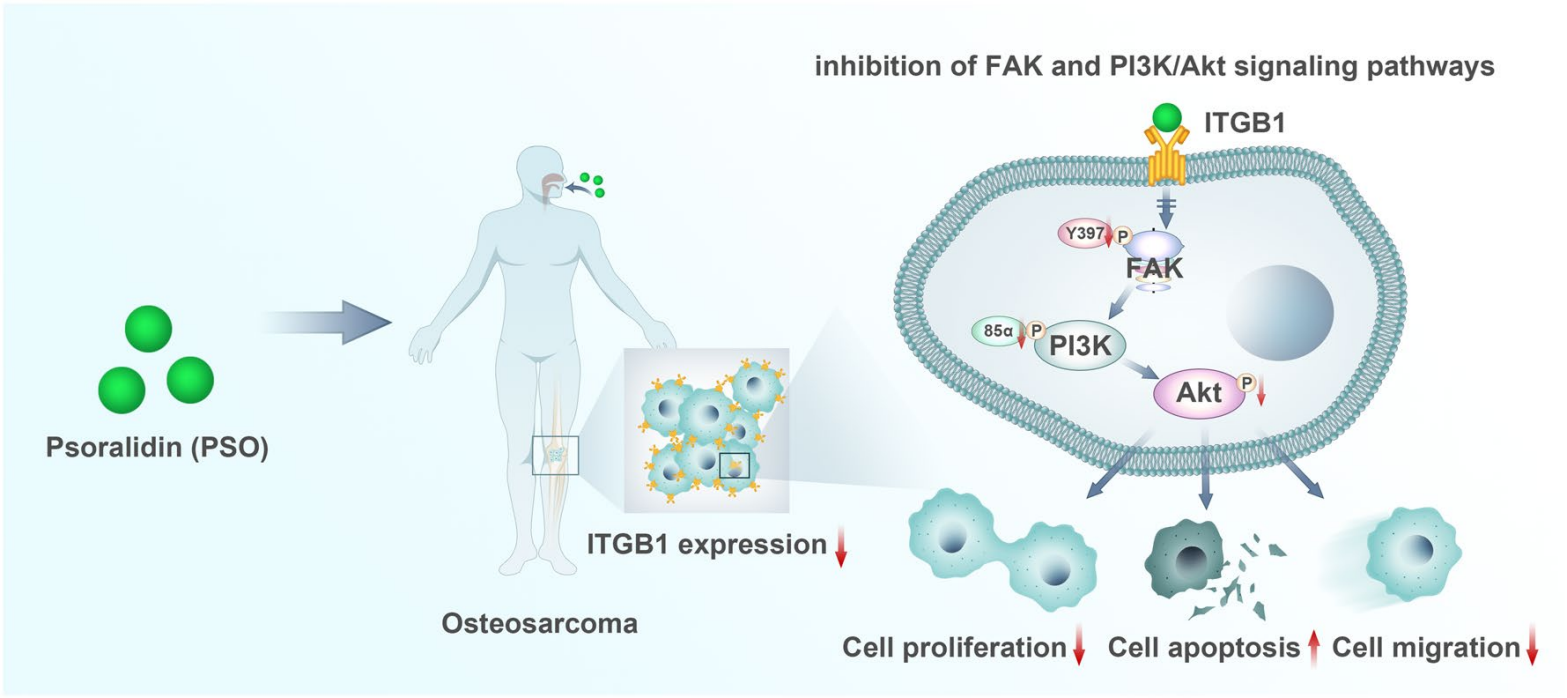
Model of the inhibitory effect of PSO on osteosarcoma functions. PSO can readily associate with ITGB1, thus affecting the signaling pathway downstream of ITGB1. ITGB1 is a transmembrane receptor that mediates the link between cells and their external environment. It mainly mediates the adhesion between cells and extracellular matrix components. Targeting ITGB1 in OS could reduce the proliferation and migration ability of OS and induce the apoptosis of OS cells

RNA-sequencing analysis was performed to explore the potential mechanism of the antitumor effects of PSO in OS cells (Fig. 7), and the results showed significant changes in PI3K/Akt and FAK signaling in 143B and MG63 cells treated with PSO. To further explore the causes of these changes, we performed GSEA and Kaplan–Meier analysis. The results revealed the importance of DEG and ITGB1, which are commonly associated with tumors. Integrin family members are membrane receptors that participate in cell adhesion and recognition, along with a variety of other cellular processes, including embryogenesis, hemostasis, tissue repair, immune response, and tumor cell metastasis and diffusion. Most integrins bind to the actin network via talin and other proteins, leading to integrin aggregation and subsequent activation of FAK and SRC. We wish to highlight that while our findings demonstrate that PSO interacts with ITGB1 at the protein level via molecular docking, other regulatory pathways also affect ITGB1 gene expression in certain processes, including the mitochondrial pathways involved in DNA repair [34, 35]. This suggests that in addition to protein-level effects, there may be other feedback mechanisms or modes of action that influence the ultimate expression of ITGB1. These processes connect integrins to downstream signal effectors, such as the PI3K/Akt, Ras-extracellular signal-regulated kinase (Ras-ERK), and Yes-associated protein (YAP)/transcriptional co-activator with PDZ-binding motif (TAZ) pathways [36]. Numerous studies have confirmed that ITGB1 is responsible for FAK activation, integrin ligand adhesion triggers an increase in FAK tyrosine (Tyr) 397 phosphorylation, and FAK has a marked effect on tumor cell survival, migration, invasion, angiogenesis, and metastasis [37, 38]. After ITGB1 activates Tyr397 phosphorylation, FAK can form a complex with SRC, which binds to the P85 subunit of PI3K, which in turn activates downstream Akt and regulate cell proliferation [39–41]. Several studies have revealed that the repression of ITGB1 expression can attenuate tumor metastasis and invasiveness [42–44]. Therefore, to improve OS treatment, it is necessary to inhibit ITGB1 expression. Molecular docking experiments showed that PSO effectively targets ITGB1. Moreover, western blot results showed that the protein expression of ITGB1 in OS cells was reduced after PSO treatment, corroborating the findings of the molecular docking analysis. Therefore, our study suggests that PSO therapy may inhibit OS growth and progression by downregulating ITGB1 expression via the FAK and PI3K/Akt signaling pathways.

## Conclusions

In conclusion, this study suggested that psoralidin has a significant inhibitory effect on OS and may downregulate ITGB1 expression via the FAK and PI3K/Akt signaling pathways to exert its anti-OS effect. In addition, this study demonstrated that ITGB1 may be a potential therapeutic target for OS treatment. Psoralidin is an excellent candidate for the development of targeted anti-OS drugs.

## Supplementary Information

**Additional file 1: Figure S1.** (a) PSO inhibits OS cell proliferation *in vitro*. (b) The safe concentration of PSO in normal cells. **Figure S2.** PSO induces OS cell-cycle arrest at G0/G1 phase. (a–d) The effect of PSO on the cell cycle of human OS cells was detected by flow cytometry. (**P < 0.01, ****P < 0.0001, vs. the control group, n = 3). **Figure S3.** (a) Heatmaps showing differentially expressed genes in OS cells treated with PSO compared to untreated control cells. (b) Gene Ontology (GO) analysis of gene enrichment in biological process, cellular component, and molecular function. **Figure S4.** The ratio of p-Src/Src in 143B and MG63 cells were significantly decreased after 24 h of PSO treatment (*P < 0.05, **P < 0.01, ***P < 0.001, ****P < 0.0001, vs. the control group, n=3). **Figure S5.** Molecular docking of the remaining three sites. 2D and 3D molecular structures of PSO, stable complex and docking pocket of PSO with MAPK (a), PIK3CD (b), PRKCA (c). **Figure S6.** Pyrinegrin partially restored the proliferative ability of OS cells treated with PSO. **Figure S7.** (a) H&E staining of heart, liver, lung, and kidney in nude mice. (b) Liver and kidney functions of nude mice in each group. **Figure S8.** H&E staining of lung in nude mice, 21 days after tail vein injection of 143B cells. **Figure S9.** Quantitative analysis of immunohistochemistry. **Table S1.** RNA primer sequences. **Table S2.** siRNA primer sequences.

## Data Availability

The datasets used and analyzed during the current study are available from the corresponding author upon reasonable request.

## Abbreviations

PSO: Psoralidin
OS: Osteosarcoma
FAK: Focal adhesion kinase
PI3K: Phosphatidylinositol 3-kinase
DMEM: Dulbecco’s modified Eagle’s medium
FBS: Fetal bovine serum
CCK8: Cell counting Kit-8
DMSO: Dimethyl sulfoxide
TBS: Tris-buffered saline
H&E: Hematoxylin and eosin
PCNA: Proliferating cell nuclear antigen
MMP: Matrix metalloproteinase
EMT: Epithelial-mesenchymal transition
ROS: Reactive oxygen species
PBS: Phosphate-buffered saline
