# Supplementary material for: Psoralidin inhibits osteosarcoma growth and metastasis by downregulating ITGB1 expression via the FAK and PI3K/Akt signaling pathways
Source: Chin Med. 2023 Mar 31;18:34. doi: 10.1186/s13020-023-00740-w (PMC10064721; doi:10.1186/s13020-023-00740-w)

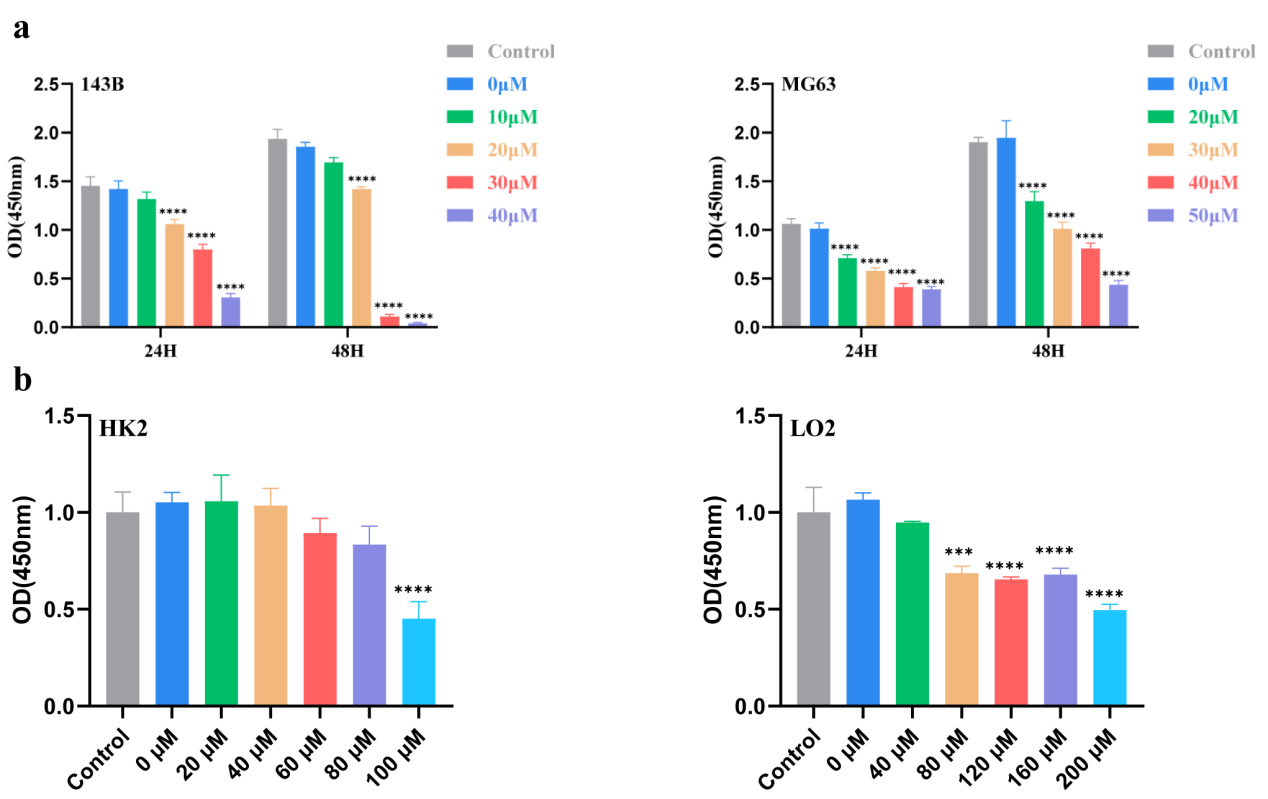
Fig. S1 (a) PSO inhibits OS cell proliferation *in vitro*. (b) The safe concentration of PSO in normal cells.


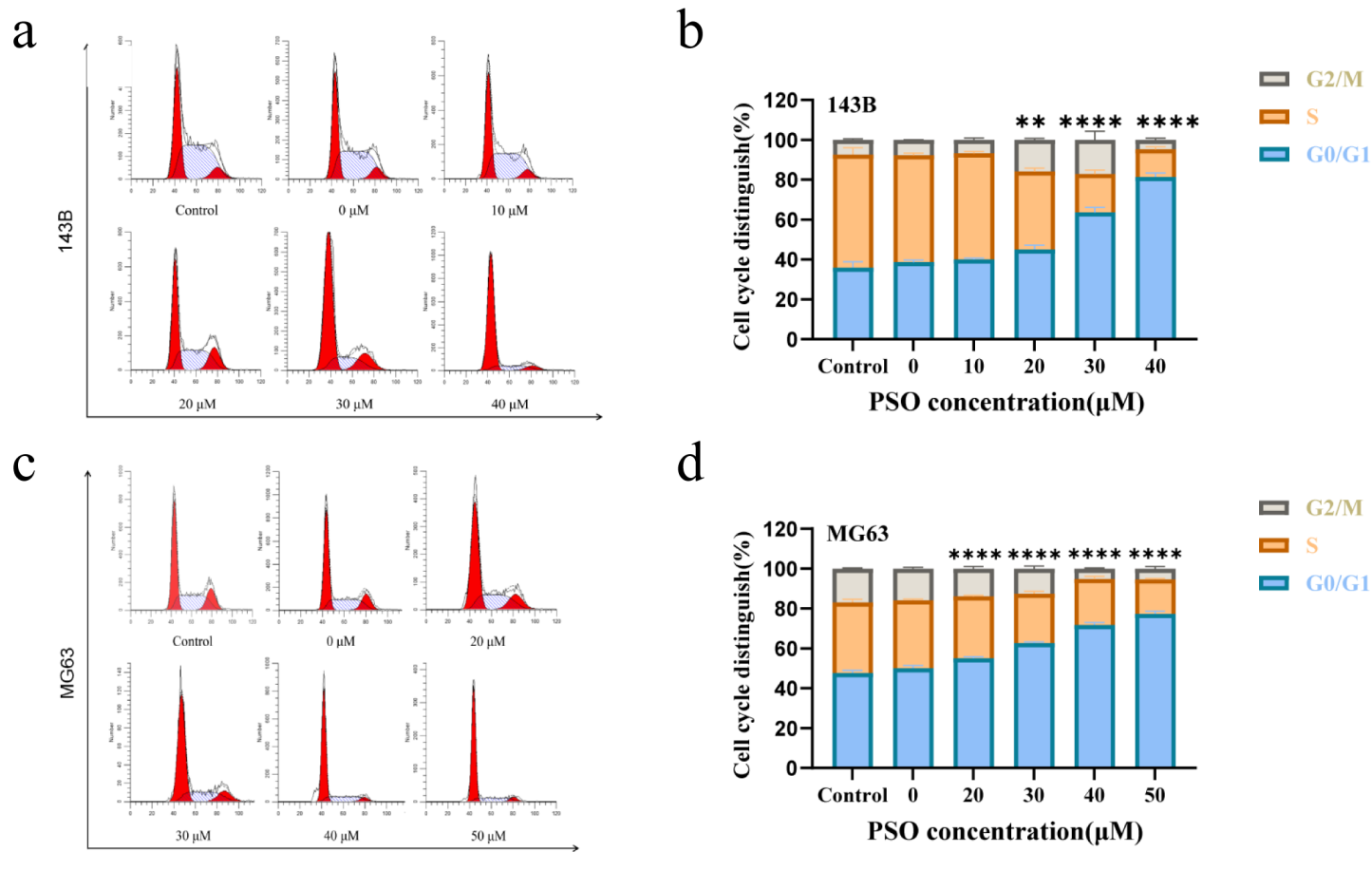
Fig. S2 PSO induces OS cell-cycle arrest at G0/G1 phase. (a–d) The effect of PSO on the cell cycle of human OS cells was detected by flow cytometry. (**P < 0.01, ****P < 0.0001, vs. the control group, n = 3).


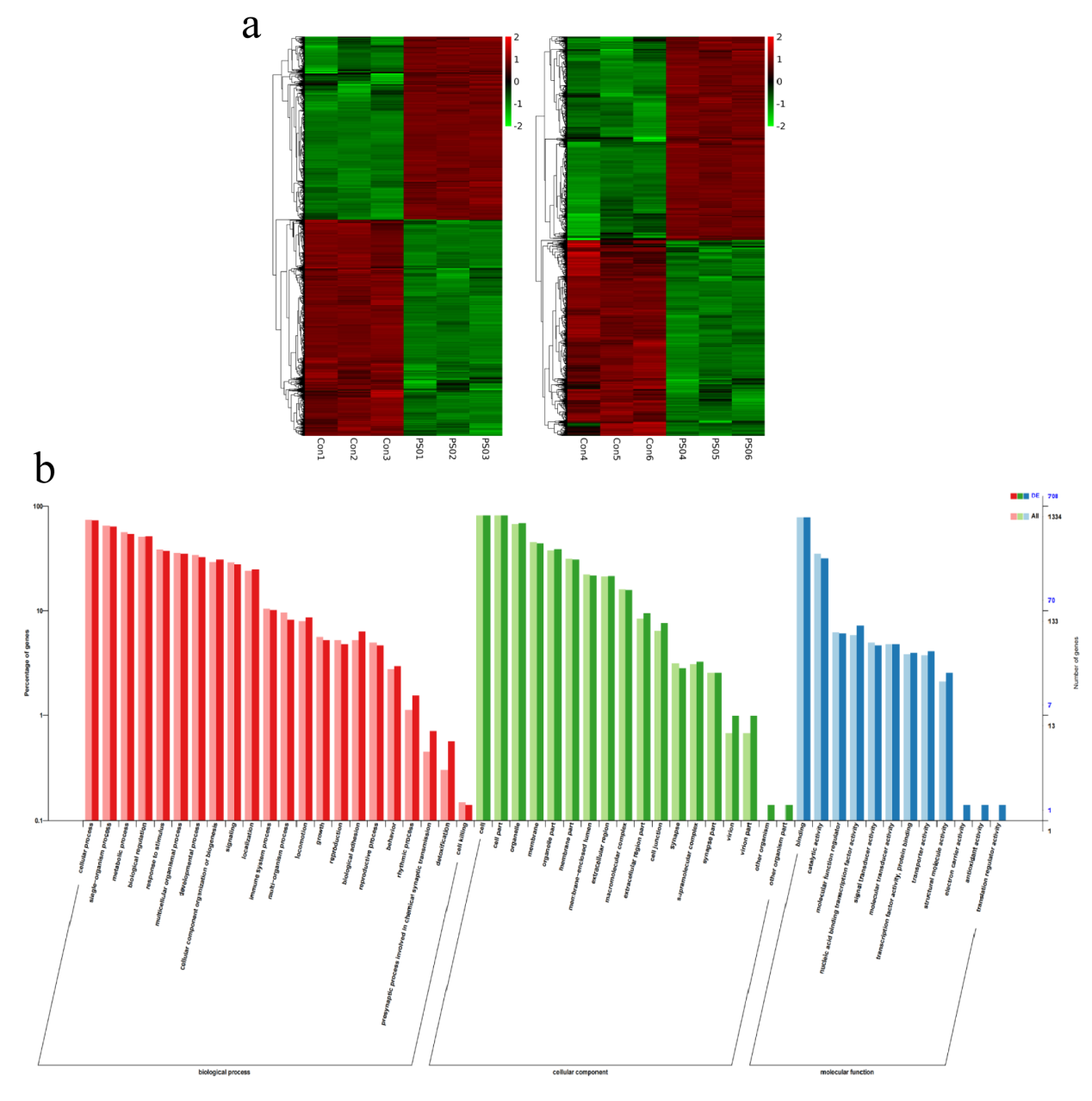


Fig. S3 (a) Heatmaps showing differentially expressed genes in OS cells treated with PSO compared to untreated control cells. (b) Gene Ontology (GO) analysis of gene enrichment in biological process, cellular component, and molecular function.


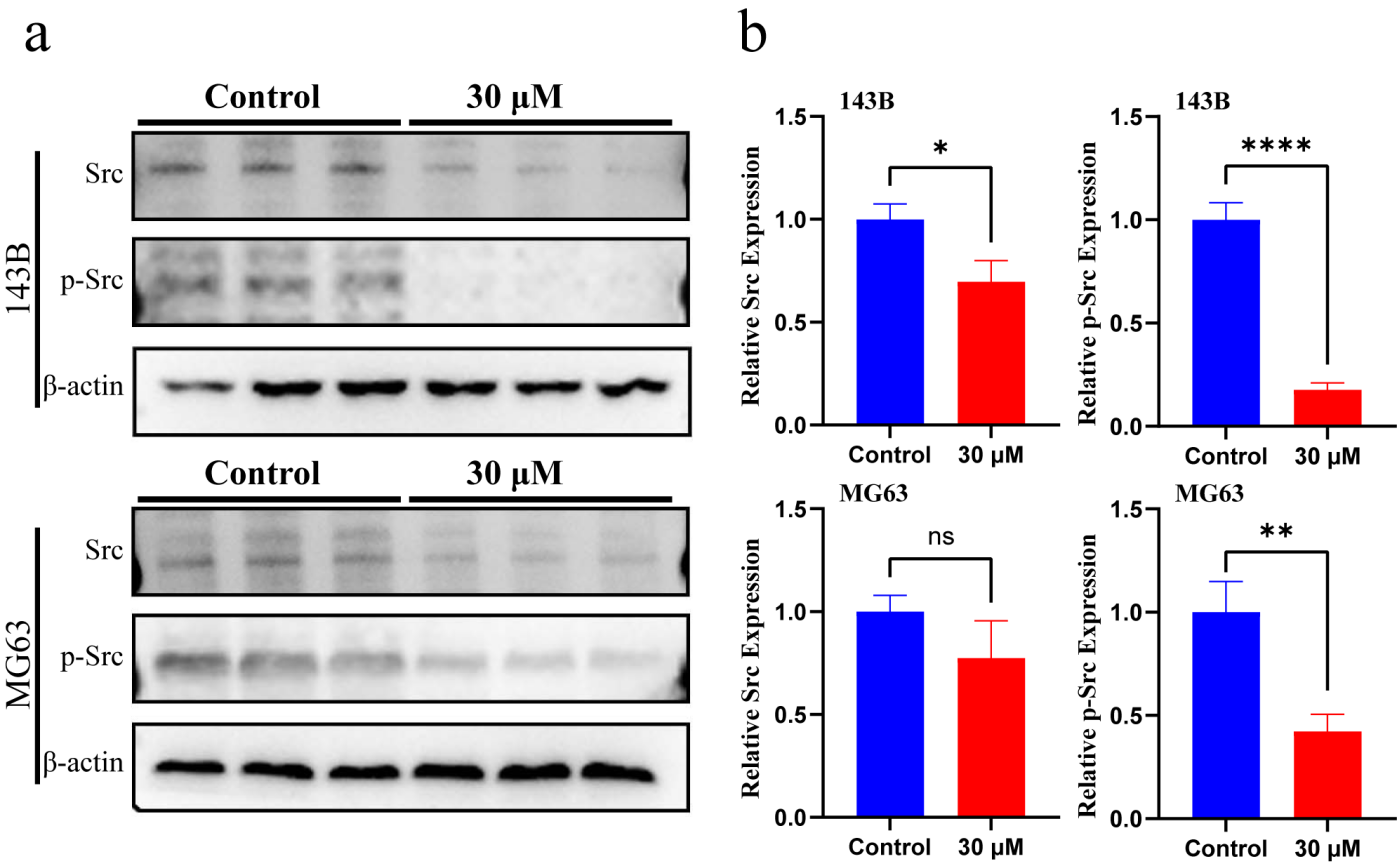


Fig. S4 The ratio of p-Src/Src in 143B and MG63 cells were significantly decreased after 24 h of PSO treatment (*P < 0.05, **P < 0.01, ***P < 0.001, ****P < 0.0001, vs. the control group, n=3).


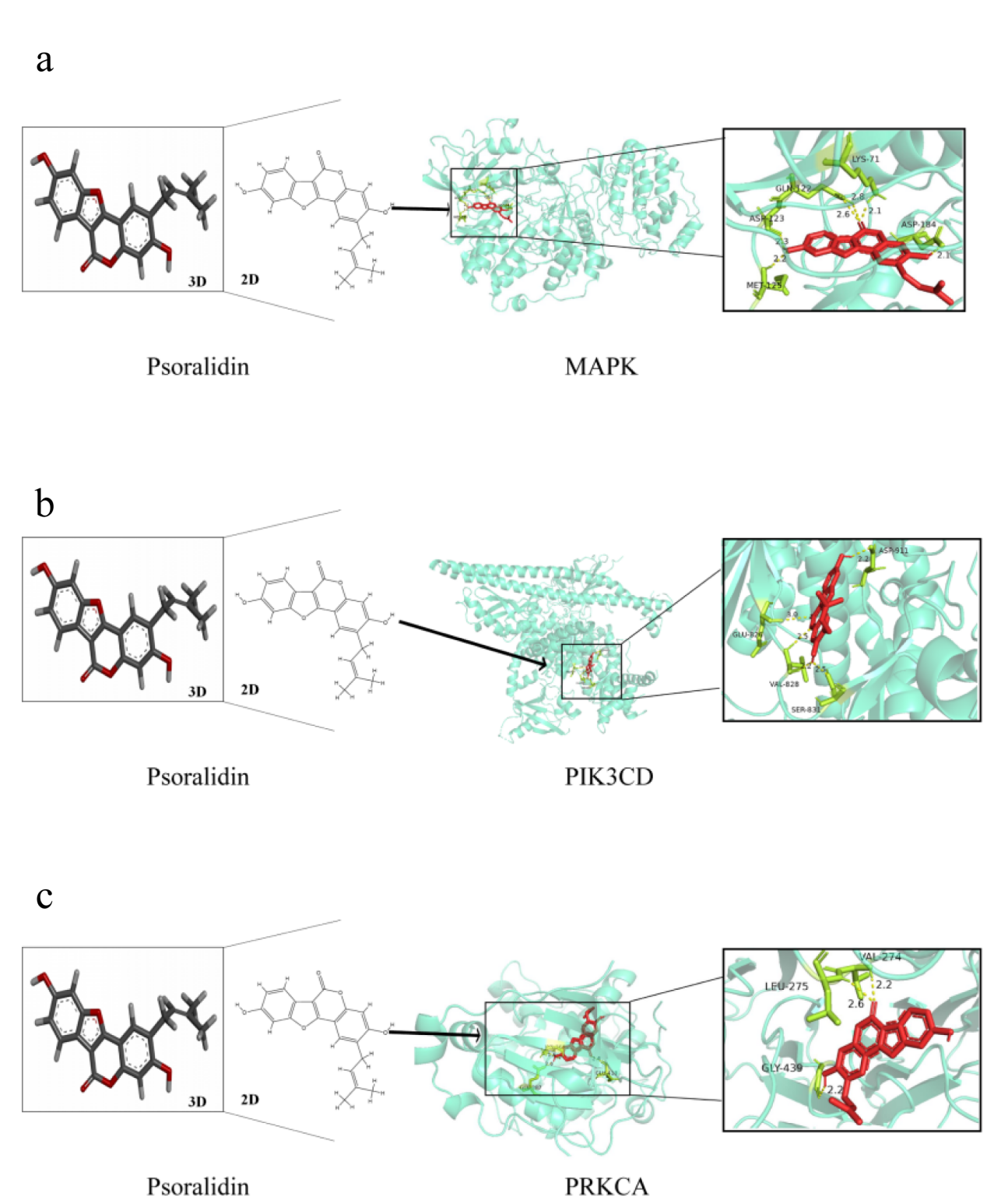


Fig. S5 Molecular docking of the remaining three sites. 2D and 3D molecular structures of PSO, stable complex and docking pocket of PSO with MAPK (a), PIK3CD (b), PRKCA (c).

Fig. S6 Pyrinegrin partially restored the proliferative ability of OS cells treated with PSO.


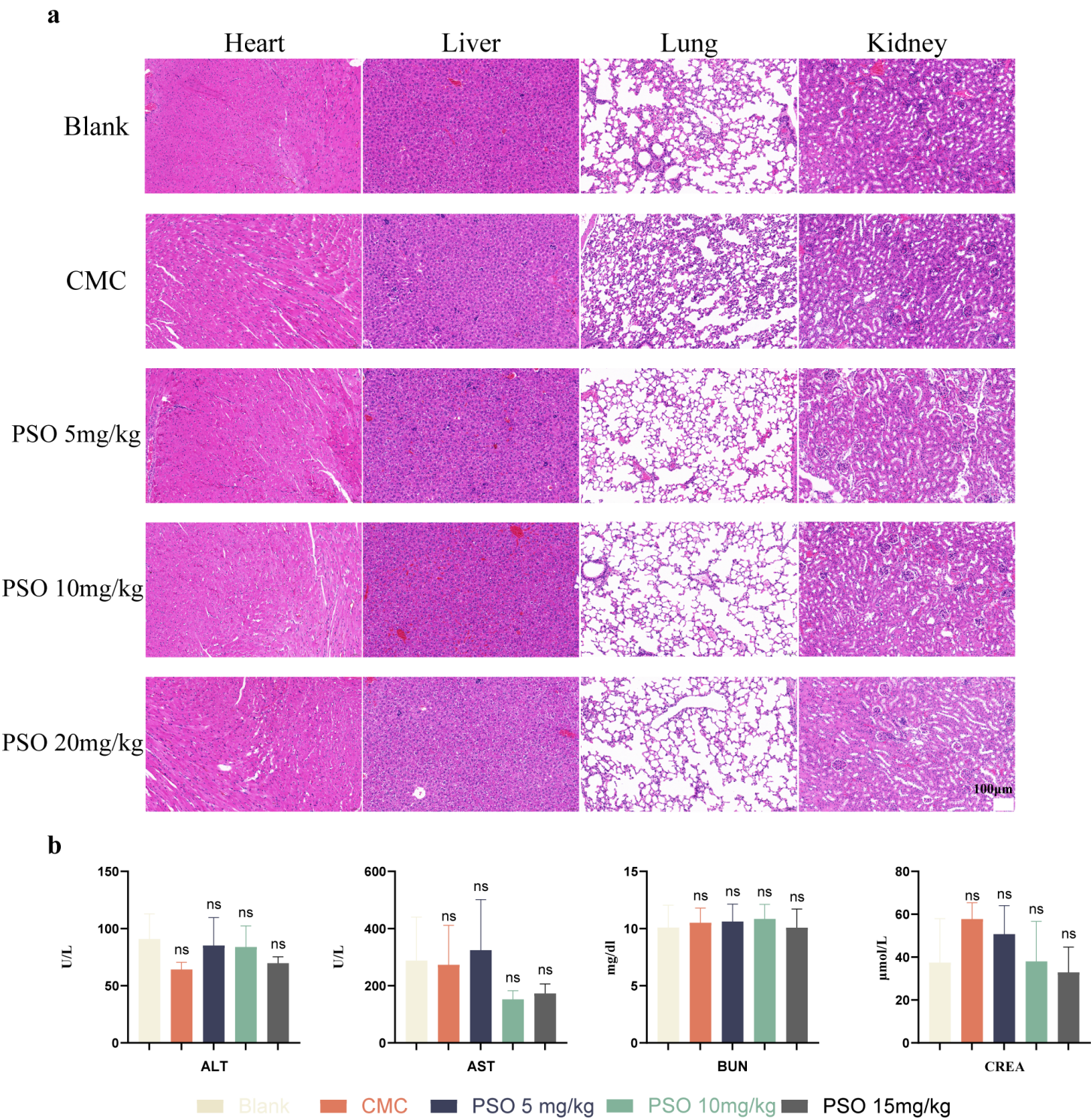


Fig. S7 (a) H&E staining of heart, liver, lung, and kidney in nude mice. (b) Liver and kidney functions of nude mice in each group.


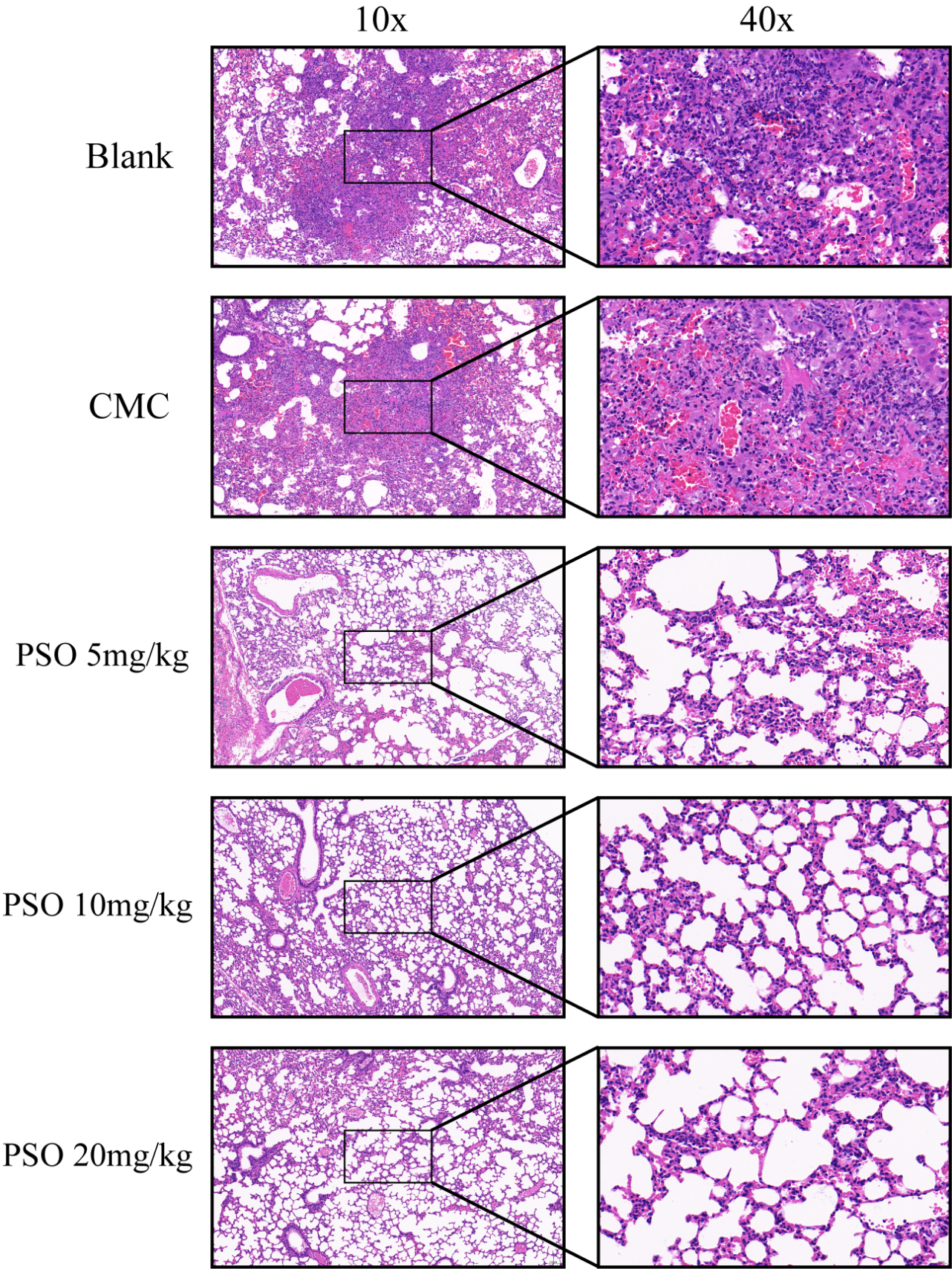
Fig. S8 H&E staining of lung in nude mice, 21 days after tail vein injection of 143B cells.


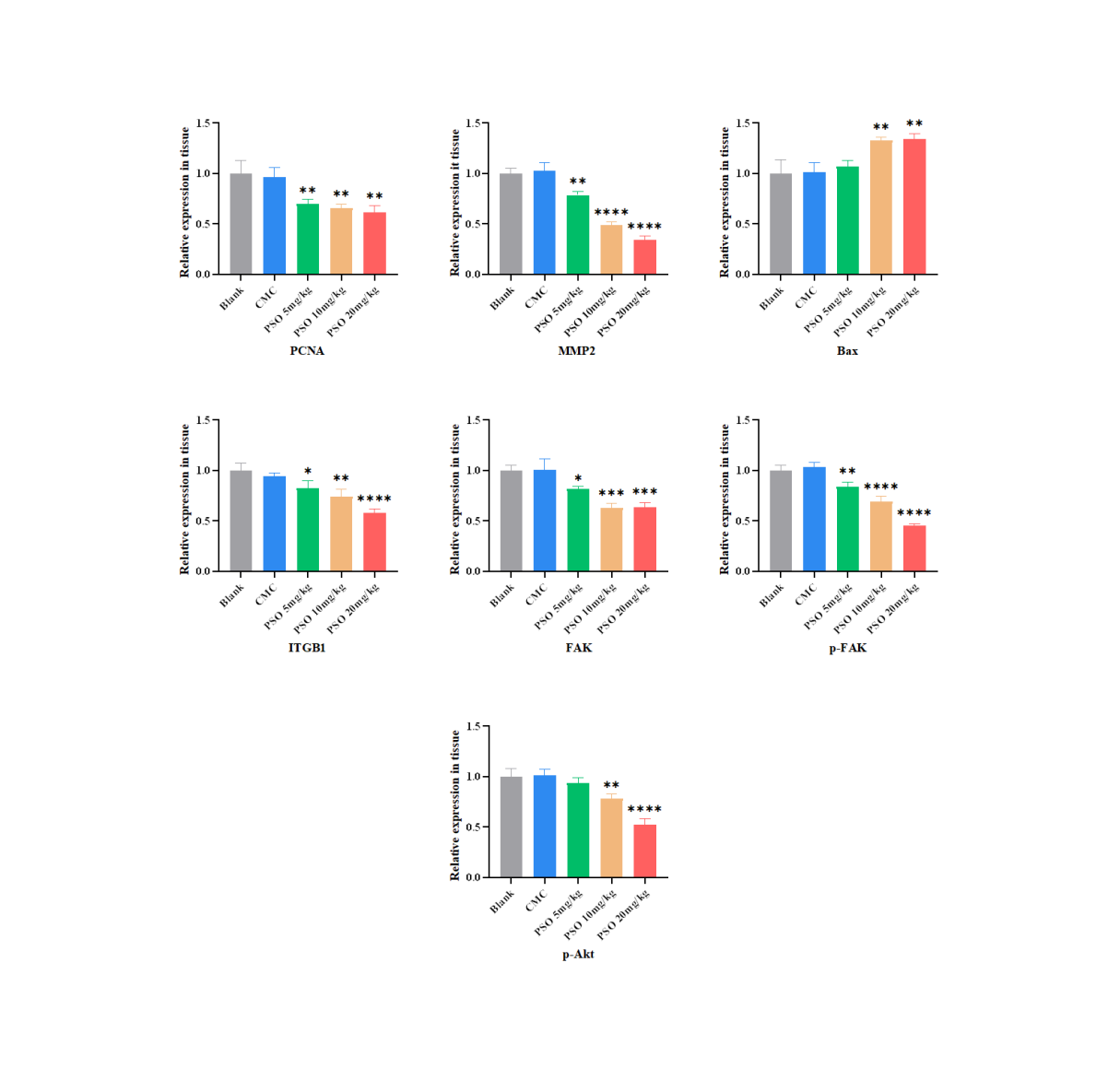


Fig. S9 Quantitative analysis of immunohistochemistry

Table S1 RNA primer sequences

| Primer | Leading chain | Trailing chain |
| --- | --- | --- |
| GAPDH | CACCATCTTCCAGGAGCGAG | TGATGACCCTTTTGGCTCCC |
| ITGB1 | GACAAATTACCCCAGCCGGT | CCAGTGGGACACTCTGGATTC |

Table S2 siRNA primer sequences
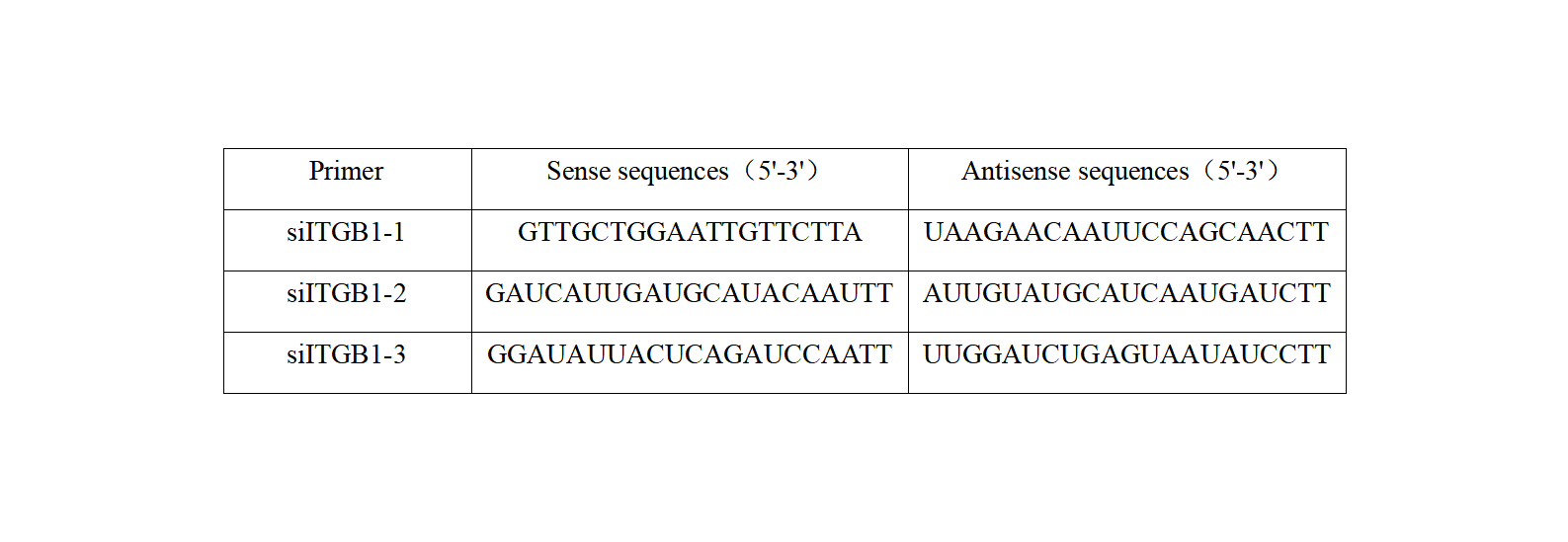

Supplement: Supplementary file 1 — Additional file 1: Figure S1. (a) PSO inhibits OS cell proliferation in vitro. (b) The safe concentration of PSO in normal cells. Figure S2. PSO induces OS cell-cycle arrest at G0/G1 phase. (a–d) The effect of PSO on the cell cycle of human OS cells was detected by flow cytometry. (**P < 0.01, ****P < 0.0001, vs. the control group, n = 3). Figure S3. (a) Heatmaps showing differentially expressed genes in OS cells treated with PSO compared to untreated control cells. (b) Gene Ontology (GO) analysis of gene enrichment in biological process, cellular component, and molecular function. Figure S4. The ratio of p-Src/Src in 143B and MG63 cells were significantly decreased after 24 h of PSO treatment (*P < 0.05, **P < 0.01, ***P < 0.001, ****P < 0.0001, vs. the control group, n=3). Figure S5. Molecular docking of the remaining three sites. 2D and 3D molecular structures of PSO, stable complex and docking pocket of PSO with MAPK (a), PIK3CD (b), PRKCA (c). Figure S6. Pyrinegrin partially restored the proliferative ability of OS cells treated with PSO. Figure S7. (a) H&E staining of heart, liver, lung, and kidney in nude mice. (b) Liver and kidney functions of nude mice in each group. Figure S8. H&E staining of lung in nude mice, 21 days after tail vein injection of 143B cells. Figure S9. Quantitative analysis of immunohistochemistry. Table S1. RNA primer sequences. Table S2. siRNA primer sequences. [file 13020_2023_740_MOESM1_ESM.docx]
